# Protein Requirements of *Oncorhynchus mykiss* Cultured in the Convection-Water Cages by Evaluating Growth, Body Composition and Liver Health

**DOI:** 10.3390/foods12010175

**Published:** 2023-01-01

**Authors:** Wei Zhao, Yu-Cai Guo, Rong Yao, An-Qi Chen, Bao-Yang Chen, Jin Niu

**Affiliations:** State key Laboratory of Biocontrol, Guangdong Provincial Key Laboratory for Aquatic Economic Animals and Southern Marine Science and Engineering Guangdong Laboratory (Zhuhai), School of Life Sciences, Sun Yat-Sen University, Guangzhou 510275, China

**Keywords:** trout, protein, antioxidation property, immune function, liver morphology

## Abstract

The diet formulation for trout has changed dramatically over the last decade due to changes in the ingredient markets and advances in feed processing technology. The protein requirements of *Oncorhynchus mykiss* were established at the end of the last century, and it is unclear whether these requirements are applicable to modern dietary formulations. Therefore, an eight-week feeding trial was performed to measure the protein requirements of *O. mykiss* by evaluating growth, body composition, antioxidation property, innate immune response and liver morphology. The five experimental diets were prepared to contain the same levels of crude lipid (120 g/kg) and graded levels of crude protein (356.3, 383.9, 411.5, 439.2 and 466.8 g/kg). The results suggested that the growth, feed utilization and whole-body crude protein levels were significantly increased when fish were fed diets containing 439.2 and 466.8 g/kg crude protein. Meanwhile, low dietary protein levels (356.3 and 383.9 g/kg) significantly down-regulated the mRNA levels of insulin-like growth factor I, catalase, glutathione peroxidase, superoxide dismutase, complement 3 and lysozyme, and also up-regulated the insulin-like growth factor binding protein 1 as well as proinflammatory cytokine expression in the liver, including interleukin 1β, interleukin 8 and tumor necrosis factor-α. Moreover, low dietary protein levels (356.3 and 383.9 g/kg) damaged liver structure, suppressed total antioxidative capacity and increased the malondialdehyde content in liver. In conclusion, high dietary protein (439.2 and 466.8 g/kg) promoted fish growth, while low dietary protein (356.3 and 383.9 g/kg) damaged liver structure, induced oxidative stress and inflammatory responses and weakened non-specific immunity. The protein requirement of *O. mykiss* reared in the convection-water cages is no less than 439.2 g/kg for optimal growth, antioxidant and immune properties.

## 1. Introduction

Protein is crucial to the growth and metabolism of fish because it provides amino acids for tissue renewal and synthesis of body protein, and is also the main component of metabolically active substances (enzymes and antibodies). In intensive aquaculture, feeds account for nearly 50–60% of the total production costs [1]. Protein is the most expensive macronutrient in fish feed, such that the economic benefits of fish production are closely related to dietary protein levels. Both excessive and inadequate dietary protein levels would inhibit fish growth and suppress immune functions and antioxidant properties [2,3,4]. Furthermore, excess dietary protein would be broken down into energy, thereby increasing ammonia emissions and reducing the quality of discharged aquaculture tailwater [2,5]. Accordingly, it is beneficial to investigate the dietary protein requirements of farmed fish for the purpose of saving breeding costs and formulating environment-friendly feed.

Rainbow trout (*Oncorhynchus mykiss*) is an important commercially farmed cold-water fish in the worldwide with an annual production of 959,600 ton in 2020 [6]. Some studies have reported the protein requirements of *O. mykiss* by evaluating growth rate and feed utilization [2,7]. The protein requirement of *O. mykiss* cultured in an indoor flow-through freshwater system ranges from 36% to 48% [7], whereas that cultured in an indoor flow-through seawater system ranges from 40–45% [2]. In addition, protein requirements vary with the size and growth stage of the fish, with juveniles requiring more protein than larger trout [8]. In previous studies, the protein requirement of *O. mykiss* was assessed with a fixed dietary lipid level of about 20% [2,9]. However, a high dietary lipid level is not conducive to feed production and preservation, and reduces the diameter and number of white muscle fibers, which affects the taste and quality of fish fillets [10]. Furthermore, the diet formulation of trout has changed considerably over the last decade due to changes in the ingredient markets, advances in feed processing technology and the emergence of faster-growing strains of trout. Therefore, it is necessary to assess the protein requirements of *O. mykiss* trout under a moderate-fat level and modern feed ingredients, which is beneficial to improve the quality of fish fillets and the market price of the product. In addition, the effects of dietary protein level on liver morphology, oxidation resistance and immune response of *O. mykiss* cultured in the convection-water cages remain unknown.

In this study, diets with fixed carbohydrate (100 g/kg), lipid levels (120 g/kg) and graded protein levels were used to determine the protein requirements of *O. mykiss* cultured in the convection-freshwater cages. The effects of dietary protein level on the growth performance and health of *O. mykiss* were evaluated by growth performance, body composition, antioxidant properties, immune response and liver morphology.

## 2. Materials and Methods

### 2.1. Test Diets

The five experimental diets were prepared to contain the same levels of crude lipid and carbohydrate and graded levels of crude protein (356.3, 383.9, 411.5, 439.2 and 466.8 g/kg), which were named P1, P2, P3, P4 and P5, respectively (Table 1). The experimental diet consisted of fish meal, soybean meal, black soldier fly (*Hermetia illucens*) larvae meal, soy protein concentrate, krill meal and chicken meal as protein sources; fish oil and soybean lecithin as lipid sources; and wheat flour as a carbohydrate source, supplemented with methionine, lysine and threonine to meet the nutritional requirements of *O. mykiss*. All ingredients were finely crushed and sieved through an 80-mesh sieve, and then weighed according to the amount required by the formula. All the required ingredients were mixed evenly, and then fish oil, soybean lecithin and water were added for further mixing. Finally, pellets with a diameter of 3.5 mm were obtained and dried in a room with a constant temperature (20 °C) to reduce the moisture to about 10%. All experimental diets were stored at −20 °C until used.

### 2.2. Feeding Trial

Nutrient feeding trials were conducted in a commercial fishery (Qinghai, China), and cages were placed in the upper reaches of the Yellow River (101. 0′27″ E, 36.8′22″ N). Fish adapted to the experimental environment for 2 weeks before the formal feeding trial, during which they were fed with commercial feed (41% crude protein, 24% crude lipid, Aller Aqua, Qingdao, China) twice a day (7:30 and 17:30) to satiation status. Thereafter, six hundred healthy and energetic fish with an initial weight of 15.82 ± 0.27 g were randomly distributed in 20 cages (2.8 m × 2.7 m × 2 m). Each experimental diet was randomly assigned to 4 cages at a density of 30 fish per cage. Fish were fed to satiation status twice a day for 56 days at 7:30 and 17:30. Throughout the feeding trial, water temperature ranged from 12–15 °C, and dissolved oxygen was above 6.0 mg/L. The feed intake of the fish per cage was recorded daily.

### 2.3. Sampling

After the feeding trial, all fish were deprived of feed for 24 h and then anesthetized with 20 mg L^−1^ of tricaine methanesulfonate (MS-222, Sigma-Aldrich, St. Louis, MO, USA). Subsequently, all fish per cage were counted and weighed individually to calculate growth parameters and survival rates. Five fish were randomly sampled from each cage and stored at −20 °C for proximate composition analysis of the whole body. A segment of liver tissue from another 6 fish per cage were rapidly deprived, frozen in liquid nitrogen and then stored at −80 °C for enzyme activity and gene transcription level analysis. Finally, three fish were randomly selected from each cage and a segment of liver tissue was cut with a sharp scalpel, then stored in 4% paraformaldehyde solution for liver morphology observation.

### 2.4. Chemical Analysis

Crude protein, crude lipid and moisture of both the experimental diets and the whole body were quantified according to Association of Official Analytical Chemists (AOAC) [11] methods. The carbohydrate contents of experimental diets were determined by anthrone colorimetry method [12]. The estimated energy of experimental diets was calculated based on previously reported methods [13], where the energy values of protein, lipid and carbohydrate were considered to be 16.7, 37.6 and 16.7 kJ/g, respectively.

### 2.5. Anti-Oxidative Parameters Analysis

A 10% liver homogenate was prepared under ice-cold conditions. Subsequently, the homogenate was centrifuged at 5000 rpm for 20 min at 4 °C to obtain the supernatant for analysis of malondialdehyde (MDA) content, superoxide dismutase (SOD) activity and total antioxidant capacity (T-AOC). Anti-oxidative parameters were examined using the corresponding detection kits (Jiancheng Bioengineering Institute, Nanjing, China).

### 2.6. Liver Morphology Analysis

Liver specimens immobilized in a 4% paraformaldehyde solution were dehydrated with gradient alcohol (70–95%), and then the specimens were embedded in paraffin for subsequent sectioning. Sections with a thickness of 5 microns were obtained and stained with hematoxylin and eosin. An optical microscope (Nikon Eclipse Ni-U, Tokyo, Japan) was used to capture the images and identify liver morphology.

### 2.7. Gene Expression Analysis

The total RNA of liver specimens from each cage was isolated using a RNAeasy™ Plus Animal RNA Isolation Kit (Beyotime, Shanghai, China) according to the supplier’s instructions. The integrity of the isolated RNA was examined by electrophoresis on a 1% agarose gel, and then its concentration and purity (OD 260/280) was tested using a spectrophotometer (NanoDrop 2000, Thermo scientific, Waltham, MA, USA). Reverse transcription was performed using a reagent kit (PrimeScript RT Reagent kit with gDNA Eraser, TaKaRa, Dalian, China), following the instructions. Likewise, the concentration and purity of cDNA were detected using a NanoDrop spectrophotometer, and then the cDNA was diluted to the same concentration with diethylpyrocarbonate (DEPC) water for the following real-time quantitative PCR. The transcription level of the target gene was measured according to a procedure mentioned in previous research [14]. The relative mRNA expression level of the target gene was calculated based on the 2^−ΔΔCT^ method, where β-actin was set as internal control gene. The gene-specific primers are shown in Table 2.

### 2.8. Calculations and Statistical Analysis

The weight gain rate (WGR), specific growth ratio (SGR), feed conversion ratio (FCR) and survival rate (SR) were calculated according to the equation previously reported [15].

The data were showed as means ± standard error (SE), and were subjected to one-way ANOVA using SPSS26.0. The data were evaluated for homogeneity and normality by the Levene’s test and the Kolmogorov–Smirnov test, respectively. Duncan’s multiple range test was used to compare whether the effect of dietary protein level on experimental parameters was significant. In addition, all data were compared by orthogonal polynomials to confirm whether there were linear or quadratic effects of dietary protein levels on experimental parameters. *p* < 0.05 was considered statistically significant.

## 3. Results

### 3.1. Biological Performance

The SR, feed utilization (FCR) and growth parameters (final body weight (FBW), WGR and SGR) are given in the Table 3. FBW, WGR, SGR and FCR were remarkedly affected by dietary protein levels, and showed both linear and quadratic effects (*p* < 0.05). The growth parameters of fish fed the P4 and P5 diets were obviously higher than those fed the other diets (*p* < 0.05), whereas a lower FCR was observed for the P4 and P5 diets (*p* < 0.05). The highest values of growth parameters and the lowest FCR were found in the P5 diet. The SR was not affected by the experimental diets (*p* > 0.05).

### 3.2. Proximate Compositions of the Whole Body

The crude protein was linearly and quadratically enhanced by dietary protein levels (*p* < 0.05) (Table 4). The fish fed the P5 diets showed the maximum crude protein, with a significant difference from that of other groups (*p* < 0.05). However, the crude lipid and moisture did not show any significant differences (*p* > 0.05).

### 3.3. Antioxidant Parameters

**Figure 1:**
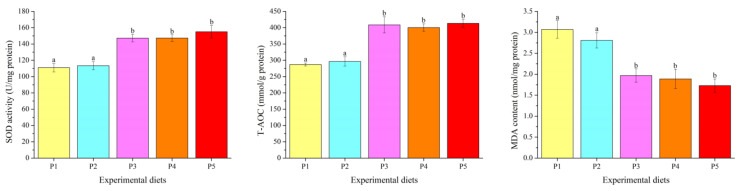
Hepatic antioxidant capacity of *Oncorhynchus mykiss* fed diets with different protein levels. Values are presented as mean ± SE, *n* = 4. The small letters indicate significant differences at *p* < 0.05. SOD: ANOVA, 0.000; linear, 0.000; quadratic, 0.000. T-AOC: ANOVA, 0.000; linear, 0.000; quadratic, 0.000. MDA: ANOVA, 0.000; linear, 0.000; quadratic, 0.000.

Experimental diets remarkedly influenced the MDA content, SOD activity and T-AOC in the liver, and also showed linear and quadratic effects (*p* < 0.05) (Figure 1). The fish fed the P3, P4 and P5 diets showed higher SOD activity and T-AOC, as well as lower MDA content, than those fed the P1 and P2 diet (*p* < 0.05).

### 3.4. Morphological Observation of the Liver

**Figure 2:**
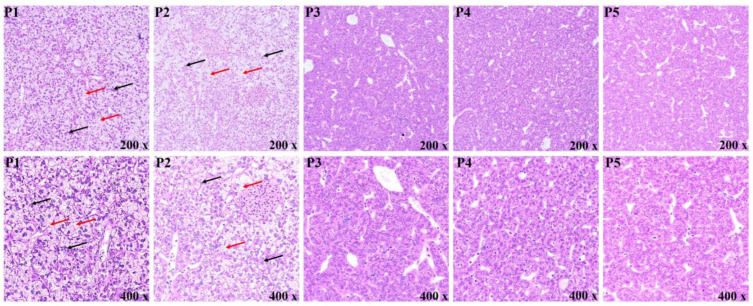
Effect of protein levels on hepatic morphology of *Oncorhynchus mykiss*. The red arrow indicates hepatocyte vacuolation. The black arrow indicates hepatocyte nuclear migration. Magnification: 200× and 400×.

The hepatocytes of the P3, P4 and P5 diets were regular in shape and closely arranged, with nuclei located in the center, and no obvious pathological symptoms were observed. However, hepatocytes of P1 and P2 diets contained numerous vacuoles of varying sizes, and nuclear migration occurred (Figure 2).

### 3.5. Genes Transcription Levels

**Figure 3:**
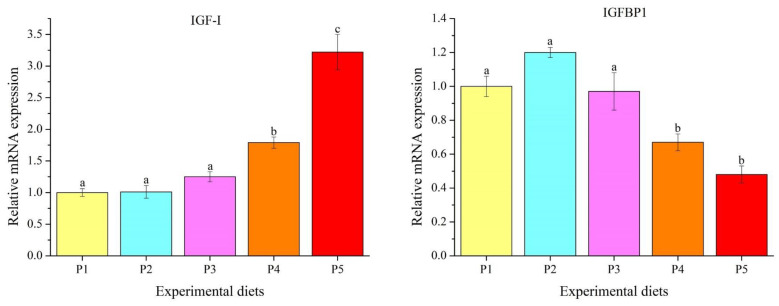
The mRNA levels of growth-related genes in the livers of *Oncorhynchus mykiss* fed experimental diets. Values are presented as mean ± SE, *n* = 4. Means with different superscripts are significantly different (*p* < 0.05). IGF-I: ANOVA, 0.000; linear, 0.000; quadratic, 0.000. IGFBP1: ANOVA, 0.000; linear, 0.000; quadratic, 0.000.

The transcription levels of the growth-related genes in the liver were markedly influenced by dietary protein levels (*p* < 0.05) (Figure 3). Insulin-like growth factor I (*IGF-I*) expression increased linearly and quadratically with the increase in the protein level, whereas insulin-like growth factor binding protein 1 (*IGFBP1*) expression decreased linearly and quadratically. P4 and P5 diets significantly up-regulated *IGF-I* expression and down-regulated *IGFBP1* expression compared to those fed the P1, P2 and P3 diets (*p* < 0.05).

**Figure 4:**
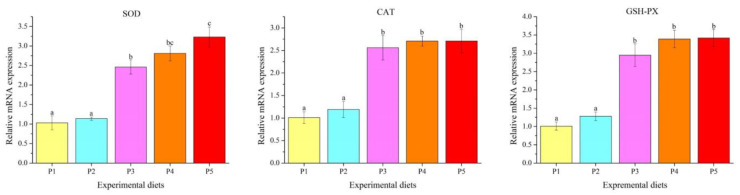
The mRNA levels of antioxidant-related genes in the liver of *Oncorhynchus mykiss* fed experimental diets. Values are presented as mean ± SE, *n* = 4. Means with different superscripts are significantly different (*p* < 0.05). SOD: ANOVA, 0.000; linear, 0.000; quadratic, 0.000. CAT: ANOVA, 0.000; linear, 0.000; quadratic, 0.000. GSH-PX: ANOVA, 0.000; linear, 0.000; quadratic, 0.000.

The transcription levels of antioxidant-related genes in the liver are given in Figure 4. Catalase (*CAT*), glutathione peroxidase (*GSH-PX*) and *SOD* expressions increased linearly and quadratically with the increase in protein levels. Fish fed the P3, P4 and P5 diets showed significantly higher transcription levels of *CAT*, *GSH-PX* and SOD than those fed the P1 and P2 diets (*p* < 0.05).

**Figure 5:**
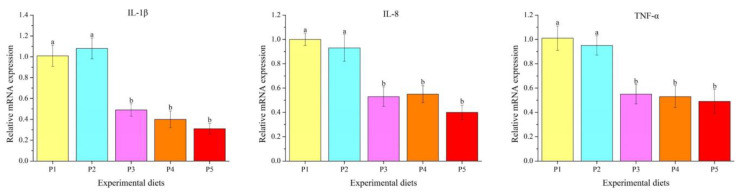
The mRNA levels of inflammation-related genes in the gut of *Oncorhynchus mykiss* fed experimental diets. Values are presented as mean ± SE, *n* = 4. Means with different superscripts are significantly different (*p* < 0.05). IL-1β: ANOVA, 0.000; linear, 0.000; quadratic, 0.000. IL-8: ANOVA, 0.001; linear, 0.000; quadratic, 0.000. TNF-α: ANOVA, 0.003; linear, 0.000; quadratic, 0.001.

Dietary protein levels obviously affected the transcription levels of inflammation-related genes in the liver (Figure 5) (*p* < 0.05). The transcription abundances of interleukin 1β (*IL-1β*), interleukin 8 (*IL-8*) and tumor necrosis factor-α (*TNF-α*) decreased linearly and quadratically with the increasing protein levels. The P3, P4 and P5 diets markedly down-regulated *IL-1β*, *IL-8* and *TNF-α* transcription abundances compared to those fed the P1 and P2 diets (*p* < 0.05).

**Figure 6:**
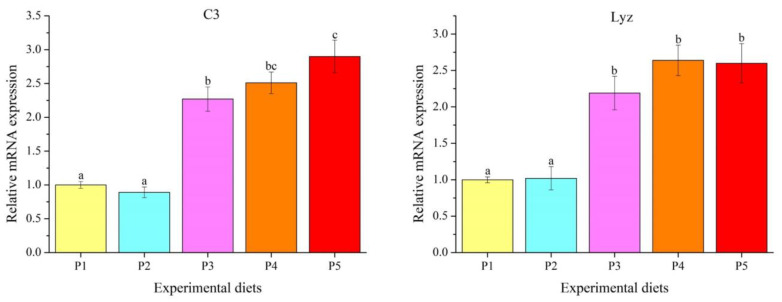
The mRNA levels of immune-related genes in the livers of *Oncorhynchus mykiss* fed experimental diets. Values are presented as mean ± SE, *n* = 4. Means with different superscripts are significantly different (*p* < 0.05). C3: ANOVA, 0.000; linear, 0.000; quadratic, 0.000. Lyz: ANOVA, 0.000; linear, 0.000; quadratic, 0.000.

Dietary protein levels had a significant effect on transcription levels of immune-related genes in the liver (Figure 6) (*p* < 0.05). Complement 3 (*C3*) and lysozyme (*Lyz*) transcription abundances increased linearly and quadratically with the increase in protein levels. Compared with the P1 and P2 diets, *C3* and *Lyz* expressions were obviously elevated in the P3, P4 and P5 diets (*p* < 0.05).

## 4. Discussion

### 4.1. Growth and Feed Utilization

In this study, the WGR and SGR showed linear and quadratic increases with increasing dietary protein levels, and the optimal growth rate was obtained when the crude protein level was 466.8 g/kg. Lesiow et al. [16] indicated that a diet containing 360 g/kg crude protein and 160 g/kg crude lipid could meet the nutritional requirements of *O. mykiss* cultured in an indoor circulating freshwater system. Moreover, a previous study suggested that the optimal crude protein and crude lipid requirements of *O. mykiss* in an indoor flow-through system ranged from 400–450 g/kg and 150–200 g/kg, respectively [17]. Seemingly, the protein requirement of *O. mykiss* in outdoor convection-water cages was higher than that of those in indoor flow-through system, which may be attributed to the fact that fish in outdoor convection-water cages require more energy for movement and to cope with various environmental stresses. The findings of this study demonstrate that a diet with a crude lipid level of about 120 g/kg and a crude protein level of less than 439.2 g/kg cannot meet the nutritional requirements of *O. mykiss* reared in outdoor convection-water cages, and the optimal protein requirement is at least 466.8 g/kg. Likewise, Ahmed and Ahmad [18] suggested that a diet with a fixed crude lipid (140 g/kg) and 450–471 g/kg crude protein level was recommended for freshwater-farmed *O. mykiss* with an initial weight of 1.56 ± 0.22 g, in order to obtain the best growth rate. Differences in the protein requirements of *O. mykiss* can be attributed to differences in dietary lipid levels, growth stages, protein sources and farming conditions (salinity, temperature and farming systems).

The FCR reflects the efficiency of fish using feed for growth purposes. In this study, lower FCR values were found at dietary protein levels below 439.2 g/kg, consistent with trends in growth performance. These findings are consistent with those of previous studies on *O. mykiss* [2,18], where optimal dietary protein levels significantly improved growth performance and inhibited FCR, whereas excess or deficiency resulted in poor growth and FCR. Similar phenomena were also reported in genetically improved farmed tilapia [3], *Misgurnus anguillicaudatus* [19] and *Argyrosomus regius* [20]. The increase in fish growth caused by optimal dietary protein may be partly attributed to the improvement of nutrient utilization.

IGF-I, a peptide hormone, is produced primarily by the liver and is involved in the regulation of cell proliferation, differentiation, growth and apoptosis [21]. IGF-I is regulated by nutritional status and nutrient metabolism, thereby affecting the growth and development of vertebrates [3]. IGF-I activation is regulated by membrane receptors for insulin-like growth factors, and IGFBPs can suppress or boost IGF-I activity by modulating IGF-I receptor availability in target tissues, primarily in liver tissues [21,22]. IGFBP1, one of the major IGFBPs, is able to prevent IGF-I from interacting with its receptor to suppress IGF-I activity in fish [21,23]. In this study, hepatic *IGF-I* expression was remarkedly up-regulated in the diets with 439.2 and 466.8 g/kg protein levels, while *IGFBP1* expression showed the opposite trend. The findings are consistent with those of WG and SGR in this study. Previous studies have shown that the high expression of *IGFBP1* in the liver inhibited the growth-promoting actions of the GH/IGF axis and led to the poor growth of *O. mykiss* [24,25]. Likewise, Liu et al. [2] suggested that the optimal protein level significantly improved growth performance and hepatic *IGF-I* expression, and inhibited hepatic *IGFBP1* expression in *O. mykiss*. Gao et al. [26] also reported that inadequate dietary protein inhibited *IGF-I* expression and caused poor growth in *Epinephelus lanceolatus*. These results suggest that optimal dietary protein could boost the growth performance of *O. mykiss* by promoting hepatic *IGF-I* expression and inhibiting hepatic *IGFBP1* expression.

### 4.2. Proximate Composition of the Whole Body

Fish growth depends on the deposition of nutrients in the body tissues, especially protein, which is the primary component of the dry-weight basis of flesh and the whole body [27]. In this study, the whole-body crude protein levels in the 439.2 g/kg and 466.8 g/kg dietary crude protein feeding groups were higher than those in the other groups, which was consistent with the results of WG and SGR. Therefore, inadequate dietary protein results in low protein deposition in the whole body, which, in turn, leads to poor growth. Likewise, Gao et al. [26] reported that optimal dietary protein could boost the whole-body crude protein level and growth performance of *E. lanceolatus*, while the dietary protein level has less effect on the whole-body crude lipid content. Liu et al. [2] also reported that optimal dietary protein significantly improved the growth performance of *O. mykiss*, which can be partly attributed to protein and lipid deposition in the whole body. A similar phenomenon has been reported in genetically improved farmed tilapia and *Caranx ignobilis*, where dietary protein levels affect protein deposition in the whole body and, thus, affect fish growth [3,28]. The effect of dietary protein levels on the whole-body protein content is dose-dependent, and the growth rate of fish reaches its maximum when body protein content is at its maximum [29]. The findings of this study suggest that optimum dietary protein levels promote protein synthesis in fish tissues, which contributes to the improvement of fish growth.

### 4.3. Liver Morphology and Inflammatory Response

Tissue morphology can directly show the health status of fish. In this study, low dietary protein (356.3 and 383.9 g/kg) resulted in hepatocyte vacuolation and nuclear migration. Likewise, Liu et al. [30] indicated that inadequate dietary protein resulted in abnormal liver morphology in *Oreochromis niloticus*, including enlargement of the area of vacuolation and nuclear migration. Sun et al. [31] also reported that insufficient dietary protein led to hepatocyte vacuolization, while excessive dietary protein resulted in enlarged hepatocytes, steatosis and formation of a large number of lipid droplets in *Aristichthys nobilis*. The vacuoles are mainly composed of lipids and glycogen, and the increase in its quantity and area is closely related to the liver’s abnormal metabolism [32]. The vacuolation of hepatocytes and liver injury caused by low dietary protein may be attributed to abnormal metabolism [33]. The liver plays a key role in lipid homeostasis, and nutritional restriction changes the structure of the liver, leading to abnormal lipid metabolism and thus disrupting lipid homeostasis [34]. Abnormal accumulation of lipids in non-adipose tissue results in cell dysfunction, which, in turn, leads to inflammatory responses and cell death [35,36]. Similar findings were observed in this study, where low dietary protein up-regulated the expression of pro-inflammatory cytokines, including *IL-1β*, *IL-8* and *TNF-α*. Likewise, nutrient deficiency- or excess-induced inflammation responses in the liver have been found in *Ctenopharyngodon idella* [37] and *A. nobilis* [31]. The findings of this study suggest that low dietary protein causes liver structural damage and further induces inflammatory responses, which may be mainly attributable to lipid deposition in the liver due to metabolic abnormalities. Metabolic abnormalities in the liver may further cause oxidative stress, which leads to poor antioxidant capacity. Therefore, we further examined antioxidant-related parameters to confirm this problem.

### 4.4. Antioxidation Property

Oxidative stress occurs when the production rate of reactive oxygen species (ROS) exceeds their removal rate, which leads to DNA damage, protein denaturation, lipid peroxidation and cell apoptosis [38]. Fish have developed two different types of antioxidant defense systems to avoid the negative effects caused by excessive ROS production, including enzymatic and non-enzymatic antioxidant systems [39]. The enzymatic antioxidant system is composed of a variety of antioxidant enzymes, including SOD, CAT and GSH-PX, and is also the first line of cellular antioxidant defense against toxicity caused by ROS [38]. SOD can eliminate superoxide by catalyzing the dismutation of O_2_^−^• into oxygen and H_2_O_2_, and CAT and GSH-PX are further responsible for converting H_2_O_2_ into water and molecular oxygen. The enzyme activity is positively correlated with the synthesis of corresponding enzyme proteins, which largely depend on gene transcription and translation [40]. In this study, low dietary protein down-regulated mRNA levels of *SOD*, *CAT* and *GSH-PX* and inhibited SOD activity in the liver. Similar phenomena have also been reported in *A. nobilis* [31], *O. mykiss* [2] and *C. Idella* [41], where dietary protein deficiency or excess reduced the activities and/or mRNA levels of antioxidant enzymes. T-AOC directly reflects the total antioxidant capacity of fish (including enzyme promoted and non-enzymatic system), while the MDA level indirectly reflects the degree of lipid peroxidation of cells. The findings of this study found that low dietary protein decreased T-AOC and increased MDA levels in the liver. This further confirms that dietary protein deficiency causes oxidative stress and weakens antioxidant properties of fish.

### 4.5. Innate Immune Response

Previous studies have suggested that dietary protein deficiency inhibited innate immune response and disease resistance in fish [31,42]. Therefore, immune-related parameters were examined to compare the effects of dietary protein levels on non-specific immunity of *O. mykiss* in this study. Lyz, a key antimicrobial protein, is able to dissolve bacteria by breaking the β-1,4-glycosidic bond between N-Acetylmuramic acid and N-Acetylglucosamine in the cell wall. Complement systems, phagocytes and lyz can play a synergistic role to boost bacteriolytic activity [43]. Accordingly, lyz and complements can evaluate the innate immune status of fish [44]. In this study, low dietary protein inhibited the *Lyz* and *C3* expression in the liver. The findings suggest that dietary protein deficiency weakens the innate immune response and reduces the production of antibacterial compounds in fish. Supporting the findings in this work, earlier studies revealed that low dietary protein had a negative effect on lyz activity, transcription levels and complement content in *A. nobilis* [31], *Labeo rohita* [42] and *C. Idella* [45].

## 5. Conclusions

In summary, fish growth is significantly promoted by increasing feed utilization and body protein deposition, as well as up-regulating *IGF-I* expression when dietary protein is not lower than 439.2 g/kg. Fish fed with a diet containing 466.8 g/kg crude protein had the best growth performance. Moreover, the diet with crude protein levels of less than 411.5 g/kg damaged liver structure, induced oxidative stress and inflammatory responses and weakened non-specific immunity. The findings of this study will provide a reference for the feed formulation of *O. mykiss* cultured in convection-water cages on the basis of modern feed materials.

## Figures and Tables

**Table 1:** Composition and nutrient levels of the experimental diets (g/kg dry matter).

Ingredients	P1	P2	P3	P4	P5
Fish meal	180	220	260	300	340
Soybean meal	170	170	170	170	170
*Hermetia illucens* meal	100	100	100	100	100
Soy protein concentrate	40	40	40	40	40
Wheat flour	110	110	110	110	110
Bone meal ^a^	156.9	119.7	82.6	45.3	8.1
Krill meal	30	30	30	30	30
Chicken meal	35	40	45	50	55
Fish oil	76.5	73.0	69.4	65.9	62.4
Soybean lecithin	20	20	20	20	20
Ca(H_2_PO_4_)_2_	10	10	10	10	10
Vitamin premix ^b^	10	10	10	10	10
Mineral premix ^c^	10	10	10	10	10
Choline	5	5	5	5	5
Vitamin C	5	5	5	5	5
DL-Met	9.1	8.3	7.5	6.7	5.9
Lys-HCL (99%)	15.5	13.2	11.0	8.8	6.6
Thr	6.9	5.7	4.4	3.2	1.9
Inositol	0.1	0.1	0.1	0.1	0.1
Sodium alginate	10	10	10	10	10
Total	1000	1000	1000	1000	1000
Nutrient levels ^d^					
Crude lipid	125.1	123.7	125.4	124.8	126.6
Crude protein	356.3	383.9	411.5	439.2	466.8
Moisture	93.2	94.9	93.8	95.1	95.2
Carbohydrate	98.5	97.9	97.3	99.8	96.8
Estimated energy (KJ/g)	12.30	12.70	13.21	13.69	14.17

^a^ Defatted and denitrified bone meal, supplied by Junyou Feed Corporation, Guangzhou, China. ^b^ Multi-vitamin (kg^−1^ diet): vitamin B1 30 mg, vitamin B2 60 mg, vitamin B6 20 mg, nicotinic acid 200 mg, calcium pantothenate 100 mg, inositol 100 mg, biotin 2.5 mg, folic acid 10 mg, vitamin B12 0.1 mg, vitamin K3 40 mg, vitamin A 3 mg, vitamin D3 0.05 mg, vitamin E 160 mg. ^c^ Multi-mineral (kg^−1^ diet): MgSO_4_∙7H_2_O 1090 mg, KH_2_PO_4_ 932 mg, NaH_2_PO_4_∙2H_2_O 432 mg, FeC_6_H_5_O_7_∙5H_2_O 181 mg, ZnCl_2_ 80 mg, CuSO_4_∙5H_2_O 63 mg, AlCl_3_∙6H_2_O 51 mg, MnSO_4_∙H_2_O 31 mg, KI 28 mg, CoCl_2_∙6H_2_O 6 mg, Na_2_SeO_3_∙H_2_O 0.8 mg. ^d^ Measured values.

**Table 2:** Primer information of real-time fluorescent quantitative PCR.

Gene	Primer Sequence (5′ to 3′)	Genbank No.
*SOD*-F	TGAAGGCTGTTTGCGTGCTGAC	NM_001160614.1
*SOD*-R	CCGTTGGTGTTGTCTCCGAAGG	
*CAT*-F	CCGTCCTTCGTCCACTCTCAGA	XM_021564302.2
*CAT*-R	CTCGGCATCCTCAGGCTTCAAG	
*GSH-PX*-F	TCATCATGTGGAGCCCTGTCTG	AF281338.1
*GSH-PX*-R	TCTGCCTCAATGTCACTGGTCA	
*IGFBP1*-F	GGAGAAGCTGGATGAATGCC	NM_001124561.1
*IGFBP1*-R	GGTCTAGGATCCCCAGCTCTTG	
*IGF-I*-F	TGCGTCCTAACCCTGACTTCG	M95183.1
*IGF-I*-R	GCAGCACTCGTCCACAATACC	
*IL-1β*-F	ACGGTTCGCTTCCTCTTCTACA	AJ245925.2
*IL-1β*-R	GCTCCAGTGAGGTGCTGATGAA	
*IL-8*-F	GTCAGCCAGCCTTGTCGTTGT	NM_001124362.1
*IL-8*-R	CGTCTGCTTTCCGTCTCAATGC	
*TNF-α*-F	GGCGAGCATACCACTCCTCTGA	NM_001124362.1
*TNF-α*-R	AGCTGGAACACTGCACCAAGGT	
*Lyz*-F *Lyz*-R	GAAACAGCCTGCCCAACT GTCCAACACCACACGCTT	AF452171.1
*C3*-F	GGCCAGTCCCTGGTGGTTA	XM_036955530.1
*C3*-R	GGTGGACTGTGTGGATCCGTA	
*β-actin*-F	TACAACGAGCTGAGGGTGGC	AJ438158.1
*β-actin*-R	GGCAGGGGTGTTGAAGGTCT	

**Table 3:** Effects of dietary protein levels on growth performance of *Oncorhynchus mykiss*.

	Dietary Protein Levels							
	P1	P2	P3	P4	P5	ANOVA	Linear	Quadratic
IBW (g)	15.88 ± 0.36	15.58 ± 0.37	15.67 ± 0.24	15.88 ± 0.18	16.09 ± 0.21	0.743	0.416	0.390
FBW (g)	55.66 ± 1.18 ^a^	56.11 ± 0.66 ^a^	55.57 ± 0.64 ^a^	60.72 ± 1.27 ^b^	64.50 ± 1.52 ^c^	0.001	0.000	0.000
WGR (%)	253.12 ± 8.07 ^a^	259.04 ± 14.38 ^a^	255.13 ± 9.99 ^a^	286.14 ± 4.51 ^bc^	306.11 ± 12.48 ^c^	0.018	0.002	0.003
SGR (%/d)	2.25 ± 0.04 ^a^	2.28 ± 0.08 ^a^	2.26 ± 0.05 ^a^	2.41 ± 0.02 ^bc^	2.50 ± 0.05 ^c^	0.020	0.002	0.003
SR (%)	98.33 ± 1.67	99.17 ± 0.83	97.50 ± 2.50	98.33 ± 0.96	98.33 ± 0.96	0.791	0.741	0.935
FCR	1.31 ± 0.03 ^a^	1.31 ± 0.08 ^a^	1.31 ± 0.02 ^a^	1.19 ± 0.04 ^bc^	1.08 ± 0.03 ^c^	0.012	0.002	0.001

Values are presented as mean ± SE, *n* = 4. The superscript small letters in the same row indicate the significant differences at *p* < 0.05.

**Table 4:** The whole-body composition of *Oncorhynchus mykiss* fed experimental diets (% wet weight basis).

Item	Moisture	Crude Protein	Crude Lipid
P1	65.79 ± 0.44	47.90 ± 0.89 ^a^	31.07 ± 0.22
P2	64.82 ± 0.52	48.18 ± 1.12 ^a^	31.28 ± 0.43
P3	66.55 ± 1.14	48.86 ± 0.28 ^a^	31.84 ± 0.31
P4	66.15 ± 0.71	50.02 ± 0.28 ^a^	31.78 ± 0.58
P5	65.53 ± 1.67	52.64 ± 0.44 ^b^	32.03 ± 0.13
ANOVA	0.790	0.004	0.369
Linear	0.792	0.000	0.039
Quadratic	0.897	0.000	0.119

Values are presented as mean ± SE, *n* = 4. The superscript small letters in the same row indicate significant differences at *p* < 0.05.

## Data Availability

The data that support the findings of this study are available from the corresponding author upon reasonable request.
